# Research on Rotating Machinery Fault Diagnosis Based on an Improved Eulerian Video Motion Magnification

**DOI:** 10.3390/s23239582

**Published:** 2023-12-03

**Authors:** Haifeng Zhao, Xiaorui Zhang, Dengpan Jiang, Jin Gu

**Affiliations:** School of Mechanical Science and Engineering, Northeast Petroleum University, Daqing 163318, China; zhangxiaorui@stu.nepu.edu.cn (X.Z.); dengpanjiang@stu.nepu.edu.cn (D.J.); nepugujin@stu.nepu.edu.cn (J.G.)

**Keywords:** motion magnification, HSV, fault diagnosis, rotating machinery

## Abstract

Rotating machinery condition monitoring and fault diagnosis are important bases for maintenance decisions, as the vibrations generated during operation are usually imperceptible to the naked eye. Eulerian video motion magnification (EVMM) can reveal subtle changes and has been widely used in various fields such as medicine, structural analysis, and fault diagnosis, etc. However, the method has a bound relationship among three parameters: spatial wavelength, amplification factor, and displacement function, so it is necessary to adjust the parameters manually in practical applications. In this paper, on the basis of the original method, an automatic solution of spatial cutoff wavelength based on brightness is proposed. First, an input video is decomposed into image sequences, their RGB color spaces are transformed into HSV color spaces, and the Value channel image representing brightness is selected to automatically calculate the spatial cutoff frequency, and then the spatial cutoff wavelength is determined, and the motion magnification video in the specified frequency band is obtained by substituting it into the original method. Then, a publicly available video is taken as an example for simulation analysis. By comparing the time-brightness curves of the three videos (original video, motion magnification video obtained by the original method and the improved method), it is apparent that the proposed method exhibits the most significant brightness variation. Finally, taking an overhung rotor-bearing test device as the object, five conditions are set, respectively: normal, rotor unbalance, loosened anchor bolt of the bearing seat, compound fault, rotor misalignment. The proposed method is adopted to magnify the motion of the characteristic frequency bands including 1X frequency and 2X frequency. The results show that no obvious displacement is found in normal working conditions, and that the rotor unbalance fault has an overall axial shaking, the bearing seat at the loose place has an obvious vertical displacement, while the compound fault combines the both fault characteristics, and the rotor misalignment fault has an obvious axial displacement of the free-end bearing seat. The method proposed in this paper can automatically obtain the space cutoff wavelength, which solves the problem of defects arising from manually adjusting the parameters in the original method, and provides a new method for rotating machinery fault diagnosis and other fields of application.

## 1. Introduction

Rotary machines such as turbines, pumps, engines and electric machines play an important role in modern industry. When they are operated for a long time in a complex working environment, especially in high-speed rotating machinery such as aircraft engines, even a small failure may lead to serious consequences. How to reduce the downtime of rotating machinery and ensure their long-term, safe and stable operation has always been a research focus, and condition monitoring and fault diagnosis technology is an important basis for maintenance decisions.

Currently, signal processing methods such as empirical mode decomposition (EMD) [1], ensemble empirical mode decomposition (EEMD) [2,3], and wavelet transformation [4] have been well applied in rotating machinery fault diagnosis [5,6]. The traditional vibration analysis technology based on spectrum analysis has been widely used in the fault diagnosis of rotating machinery, and has formed a lot of fault spectrum features with reference values, but it needs experienced personnel to perform the analysis, and there is not a one-to-one mapping relationship between the fault condition and the spectrum characteristics, but rather a one-to-many mapping relationship, which increases the difficulty of fault diagnosis. In this field, the author often encounters the problem that it is difficult to accurately identify rotating machinery faults by using spectrum characteristics. In contrast, the operational deflection shape (ODS) analysis method [7] not only realizes fault-source localization but also visually and intuitively displays structural vibration patterns under specific working conditions (that is, at a specified time or at a specified frequency). It has achieved satisfactory outcomes in fault diagnoses in rotating machinery such as centrifugal pumps [8] and rotor systems [9,10,11]. However, the above methods require conventional vibration sensors, which not only have limited measurement points but are also difficult to install in environments with high temperatures, high pressures, the presence of oil and gas, or corrosion. Furthermore, mass load is generated on lightweight structures, which affects the vibration characteristics of equipment and reduces the accuracy of analysis results. Non-contact fault diagnosis methods offer a new approach to solving these challenges. At present, the non-contact fault diagnosis methods used in rotating machinery fault diagnosis mainly include the optical method [12,13], the three-dimensional digital image correlation (3D-DIC) [14,15,16] method, and the computer vision method [17,18,19,20]. However, the optical method based on a laser vibrometer is still mainly based on local vibration measurements, and the full-field and multi-directional vibration detection is still in the research stage. The 3D-DIC method usually requires at least two high-speed cameras, incurring higher costs and demanding strict operating environments, making practical application inconvenient. The computer vision method needs traditional signal processing technology to extract fault features, but the analysis results are not visual or intuitive, which affects the practicability of this method to a certain extent.

Motion magnification (MM) is a new method of motion detection which processes videos captured by high-speed cameras and magnifies the subtle motions invisible to the naked eye to make them obvious. The MM methods proposed in recent years are based on the Eulerian perspective [21], and the changes of each fixed pixel (or region) of the measured object in the video are observed to achieve pixel-level motion magnification. The method was originally used for medical remote monitoring. Sushma [22] adopted the method of motion magnification to detect motion in perfusion-weighted MRIs, so as to reduce the data destruction caused by patients’ involuntary activities during the wait for blood perfusion. Thomas V. et al. [23] adopted the motion magnification method based on the Riesz Pyramid to make the changes in blood flow, head movement and skin and facial color controlled by the heartbeat’s rhythm more obvious. Dionysius M. Siringoringo et al. [24] used a vision-based motion magnification method to extract the vibration of viaduct light poles, and accurately extracted the multi-modal vibration characteristics of light poles through his analysis. Eitner M. et al. [25] used the broad-band phase-based motion magnification (BPMM) method to measure nozzle vibration under pressurized air excitation and clearly identified the sixth-order mode. The results of the modal parameter estimation were significantly improved. Luo K et al. [26] proposed the measurement method of cable bridge vibration based on the broad-band phase video motion magnification (BPVMM) method. Aral Sarraf et al. [27] used the phase-based motion amplification method to identify the structural motion of wind turbine blades under the condition of damage from the captured image sequence. Liu P et al. [28] proposed an ultrasonic signal enhancement method based on phase-based motion magnification to estimate the residual fatigue life of steel plates. Fioriti V et al. [29] devoted themselves to the application of motion magnification methods in the field of cultural heritage protection and monitoring in Italy. Michał ’Smieja et al. [30] introduced some application cases of the motion magnification method in the fault diagnoses of rotating machinery base bolt loosening, coupling misalignment and shaft end-sealing defects, etc. Although the method has been studied and applied in the fields of medical treatment, structure [31], fault diagnosis, etc., the important parameters affecting the motion-magnification effect (i.e., the spatial cut-off wavelength) in the current literature are still determined by manual adjustment, which limits the practicability of the method.

Based on the analysis of the Eulerian motion magnification method, this paper introduces the concept of brightness, and proposes an automatic calculation method of spatial cutoff wavelength based on brightness, so as to obtain the motion magnification image. Then, the proposed method is used to process a publicly available video in the Ref. [32], and the time-brightness curve is used to compare the brightness changes in the original video and the motion magnification videos obtained by original methods and improved methods to verify the effectiveness of the proposed method (related videos can be found in the Supplementary Materials). Finally, taking an overhung rotor-bearing experimental device as the object, four fault conditions are set, respectively: rotor unbalance, loosened anchor bolt of the bearing seat, compound fault (rotor unbalance and loosened anchor bolt of the bearing seat), and rotor misalignment, and the characteristic frequency band including 1× and 2× frequency is magnified by the method, and the motion-image characteristics of the four fault conditions are revealed by comparing them with the normal working conditions. The proposed method can automatically obtain the spatial cutoff wavelength, solve the drawback of manual parameter adjustments in the original method, improve the efficiency, and provide a new method for rotating machinery fault diagnosis.

## 2. Methods

### 2.1. The Eulerian Motion Magnification Method

The Eulerian motion magnification method is an improvement on the traditional optical flow method [33], and its fundamental concept is that the change of brightness in each pixel in the image over time reflects the change of the pixel’s displacement [34]. The detailed derivation process of the method can be found in Ref. [32]. This paper only quotes the main steps to illustrate the three constraint parameters of the method. In this paper, the concept of motion magnification is briefly explained using an example of a one-dimensional signal undergoing translational motion. Let *I*(*x*, 0) = *f*(*x*) represent the image intensity at position *x* at time *t* = 0, and *I*(*x*, *t*) = *f*(*x* + *δ*(*t*)) represent the image intensity at position *x* at time *t*. Here, *δ*(*t*) denotes the displacement function of image pixels, signifying the pixel’s displacement from time *t* = 0 to time *t*. Perform the first-order Taylor expansion with respect to *x* on *I*(*x*, *t*) and subtract *I*(*x*, 0) to obtain:

(1) $$B\left(x,t\right)=\delta \left(t\right)\frac{\partial f\left(x\right)}{\partial x}$$

*B*(*x*, *t*) represents the displacement variation of image pixels at position *x* from time 0 to *t*. According to Equation (1), if the displacement variation of pixels is increased, the displacement function *δ*(*t*) needs to be multiplied by the amplification factor *α*. After a series of derivations, the result can be obtained,

(2) $$\tilde{I}\left(x,t\right)\approx f\left(x\right)+\left(1+\alpha \right)\delta \left(t\right)\frac{\partial f\left(x\right)}{\partial x}$$

Reverting Equation (2) back to its pre-expanded form,

(3) $$\hat{I}\left(x,t\right)=f\left(x+\left(1+\alpha \right)\delta \left(t\right)\right)$$

In Equation (2), *Ĩ*(*x*, *t*) represents the actual value of the signal after motion magnification, while Equation (3) represents the theoretical value. This indicates that during the motion magnification process, the motion of *f*(*x*) at time *t* is amplified by a factor of (1 + *α*), while also reflecting the pixel intensity change at that moment. It is worth noting that this analysis process is only illustrated using a single component signal as an example. When analyzing real signals with multiple components, filtering can be performed within the specified frequency band (*ω_l_*, *ω_h_*), while *ω_l_* and *ω_h_* denote the lower cutoff frequency and the upper cutoff frequency, respectively.

In order to solve the problem that the first-order Taylor series approximation becomes inaccurate due to the rapid change in image function *f*(*x*), so that the actual value expressed by Equation (2) is approximately equal to the theoretical value expressed by Equation (3), the boundary of the amplification factor *α* should be derived according to the spatial frequency *ω* [32]. When the spatial frequency is assumed to be *ω*, it can be derived from the law of cosines and *ω* = 2π/*λ*,

(4) $$\left(1+\alpha \right)\delta \left(t\right)<\frac{\lambda }{8}$$

Equation (4) provides the direct constraint relationship among the spatial wavelength *λ*, the amplification factor *α* and the displacement function *δ*(*t*). However, in practical applications, the motion magnification effect is affected by multiple parameters. Among them, the amplification factor *α*, the specified frequency band (*ω_l_*, *ω_h_*), and other parameters can be specified by the user or selected through prior knowledge. However, the spatial cutoff wavelength *λ_c_*, which affects the output video quality, can violate the approximation limit in order to enhance visual perception [32]. As a result, *λ_c_* is difficult to be set by experience or prior knowledge and requires manual parameter adjustment, which limits the application of the method. Therefore, it is necessary to study a method that can calculate *λ_c_* automatically to avoid human interference and improve computational efficiency. It is worth noting that if the current spatial wavelength *λ* is less than the *λ_c_* threshold, the amplification factor α will be attenuated to reduce the amplification of noise and improve the quality of the output video.

### 2.2. The Automatic Calculation Method for Spatial Cutoff Wavelength

All video images were in RGB color space, and each color appeared in its primary spectral components of red, green and blue [35]. There was a problem in the mixing of chrominance and intensity information [36], which resulted in the three color components changing with the brightness. It was difficult to extract the brightness information and it was unsuitable for image processing. Compared with RGB, HSV color space was closer to people’s perception experience of color, and could intuitively express hue, saturation and value [37]. It can be seen from Section 2.1 that the change in brightness reflected the change in the displacement of image pixels. Therefore, according to the concept of brightness, the conversion relationship between RGB color space and HSV color space was introduced to obtain the brightness value, and then the spatial cutoff wavelength was calculated in this paper. For any coordinate point in the image, its corresponding value in the RGB color space was assumed to be *R*, *G*, and *B*, and its conversion relationship is shown as follows [38],

(5) $$\begin{array}{ccc} R^{\prime }=\frac{R}{255} & G^{\prime }=\frac{G}{255} & B^{\prime }=\frac{B}{255} \end{array}$$

Equation (5) normalizes the corresponding value in the RGB color space between 0 and 255, and then sets

(6) $$C_{\max}=\max\left(R^{\prime },G^{\prime },B^{\prime }\right)$$

The compute value,

(7) $$V=C_{\max}$$

The *V* component image containing only the brightness value (i.e., Value) in HSV color space was extracted, and the spatial frequency was calculated from the row frequency and column frequency of the image. The *RF* was assumed to be the row frequency, *CF* was column frequency, and *SF* was spatial frequency. For an image F with *M* × *N*, where *M* is the number of rows and *N* is the number of columns, the *RF*, *CF* and *SF* of the pixel at the point (*m*, *n*) were calculated by Equations (8), (9) and (10), respectively [39].

(8) $$RF=\sqrt{\frac{1}{MN}{\sum_{m=0}^{M-1}\sum_{n=1}^{N-1}\left[F\left(m,n\right)-F\left(m,n-1\right)\right]}^{2}}$$

(9) $$CF=\sqrt{\frac{1}{MN}{\sum_{m=0}^{M-1}\sum_{n=1}^{N-1}\left[F\left(m,n\right)-F\left(m-1,n\right)\right]}^{2}}$$

(10) $$SF=\sqrt{{\left(RF\right)}^{2}+{\left(CF\right)}^{2}}$$

According to *ω* = 2π/*λ*,

(11) $$2\pi \times SF=\frac{2\pi }{\lambda_{c}}$$

Therefore, the spatial cutoff wavelength was

(12) $$\lambda_{c}=\frac{1}{SF}$$

The calculated spatial cutoff wavelength was used as the input parameter of the original Eulerian motion magnification method, and the video of the motion magnification could be obtained for fault diagnosis.

### 2.3. The Improved Eulerian Video Motion Magnification Method Flow

**Figure 1:**
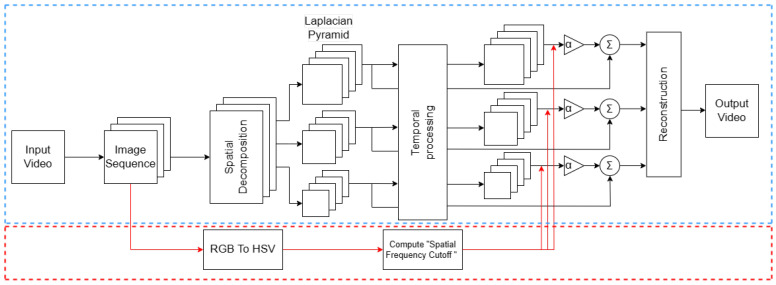
Improved Eulerian video motion magnification process.

Based on the traditional Eulerian motion magnification process, combined with the method above in Section 2.2, an improved Eulerian video motion magnification method was proposed, and its process is shown in Figure 1. The blue dashed line box in Figure 1 shows the process in Ref. [32], while the red dashed line box and arrow refer to the calculation process of the proposed spatial cutoff wavelength.

For a given input video, the Eulerian motion magnification method steps are as follows.

Step 1: Decompose the input video by frames into image sequences;
Step 2: The image sequences in step (1) are converted from RGB color space to HSV color space, and the spatial cutoff wavelength *λ_c_* is calculated;
Step 3: The image sequences in step (1) are decomposed into different spatial frequency bands [40] by Laplace pyramid and filtered by butterworth filter to obtain the image signal of the specified frequency band;
Step 4: Using the given amplification factor α and the spatial cutoff wavelength *λ_c_* calculated in step (2), the motion magnification of each image signal obtained in step (3) is carried out, which is added back to the original signal for reconstruction. Then, the motion magnification video is obtained.

### 2.4. Simulation Analysis

**Figure 2:**
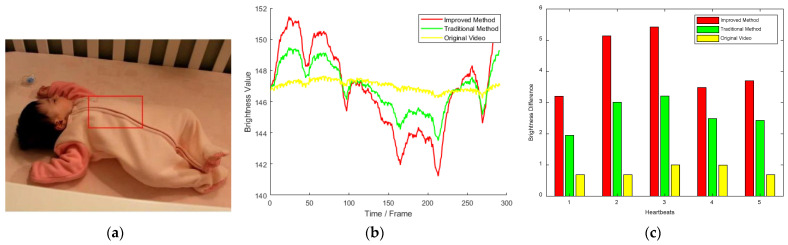
Original video frame and time-brightness curve comparison. (**a**) Original video frame (in Ref. [32]); (**b**) time-brightness curve; (**c**) the amplitude comparison of brightness fluxion.

In order to verify the effectiveness of the proposed method, the publicly available video in Ref. [32] was used for motion magnification. The original video frame is shown in Figure 2a, with a resolution of 960 × 544. The video contains several of the baby’s heartbeats, which are difficult to see with the naked eye. Using the method presented in Section 2.2, the spatial cutoff wavelength *λ_c_* = 40 was calculated. In order to compare it with the motion magnification video provided by MIT, the parameter values of the two methods were set to be the same, that is, the amplification factor *α* = 20, and the lower cutoff frequency *w_l_* and the upper cutoff frequency *w_h_* were set to 0.4Hz and 3Hz, respectively.

ImageJ software (v.1.53t) was used to extract the time-brightness curve of the heartbeat region shown by the red rectangle in Figure 2a for the original video and the processed video, respectively, as shown in Figure 2b. Where the horizontal axis was the number of frames, the vertical axis was the brightness value. The yellow represents the time-brightness curve of the unprocessed original video, the green is the time-brightness curve of the motion magnification video given by MIT, and the red is the time-brightness curve of the motion magnification video obtained by the proposed method in the paper. Figure 2c is a comparison diagram of the brightness difference corresponding to the adjacent maximum frame and minimum frame reflecting heartbeat in Figure 2b. Where the horizontal axis was the heartbeat number, the vertical axis was the brightness difference. It can be seen from Figure 2b,c that the brightness fluctuation of the original video was not obvious, the time-brightness curve fluctuation amplitude of the motion video obtained by the proposed method in the paper was significantly higher than that of MIT, and the characteristics of the baby’s heartbeat curve could be clearly represented according to the brightness change. In addition, from the comparison of computing times, under the same computer-operating environment, the running time of the method used in Ref. [32] was 43.34 s, while that of the proposed method was 41.264 s, which was very close. Therefore, the simulation analysis verified the effectiveness of the proposed method.

## 3. Experiments

### 3.1. Experimental Setup

**Figure 3:**
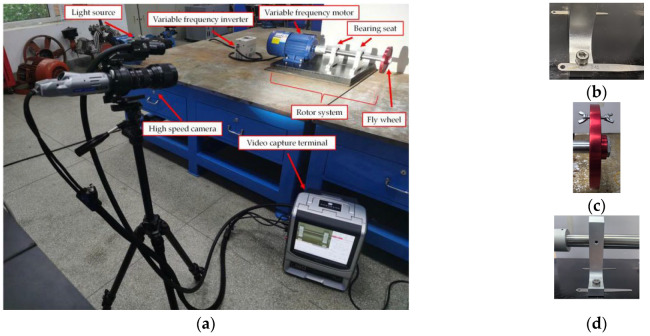
Details of the experimental setup. (**a**) Experimental setup; (**b**) condition 2; (**c**) condition 3; (**d**) condition 5.

To validate the effectiveness of the proposed method for rotating machinery fault diagnosis, an experimental setup shown in Figure 3 was constructed. 

It mainly consisted of an overhung rotor-bearing system, a no-strobe light, and a high-speed camera (KEYENCE VW-600C, Keyence, Osaka, Japan). The overhung rotor-bearing system consisted of a variable-frequency inverter, a variable-frequency motor, a drive-end bearing seat, a free-end bearing seat, a fly wheel, and a base. The motor and bearing seats were bolted on the base. A rubber pad was used for vibration isolation between the base and the table. The diameter of the shaft was 25 mm and its length was 400 mm, one end of which was connected to the motor by an elastic coupling, and the other end was installed with the fly wheel (150 mm in diameter and 15 mm thickness). Two bearing seats with built-in 6205 deep-groove ball bearings were installed at both ends of the shaft, with a spacing of 130 mm, where the drive-end was 65 mm away from the shaft end connected with the coupling. The rotational speed of the variable-frequency motor could be adjusted through the variable-frequency inverter. This paper considers five work conditions: normal (condition 1), loosened anchor bolt of the drive-end bearing seat (condition 2), rotor unbalance (condition 3), compound fault (condition 4), and rotor misalignment (condition 5). Detailed descriptions are given in Table 1.

### 3.2. Test Plan

**Figure 4:**
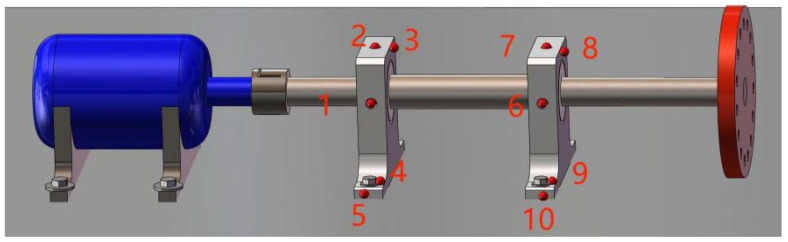
Diagram of vibration measurement points.

To compare the differences between the traditional contact fault diagnosis method and the improved Eulerian video motion magnification method, and verify the accuracy of the proposed method, a JDI930 vibrometer was first used to measure the vibration value (mm/s) at 10 measurement points (as shown in Figure 4) under five conditions. During the test, the rotor operating frequency was adjusted to 24.1 Hz, equivalent to 1446 RPM. After the vibration measurement, the captured area of the high-speed camera was adjusted through the video capture terminal, which covered the two bearing seats’ areas. Finally, the capture frame rate was set to 1000 fps, the resolution was set to 640 × 480, and the no-strobe light was turned on for video capture. Where the light source power was 60 W, the color temperature was 8000 K, and the brightness was set to the maximum through the acquisition software when the video was captured.

## 4. Results and Discussion

### 4.1. Results and Analysis of Vibration Measurements 

According to the vibration test plan in Section 3.2, the JDI930 vibrometer (Jditech, Dalian, China)was used to measure 10 measurement points of the experimental setup under five working conditions, and the measured values are shown in Table 2.

It can be seen from Table 2 that under condition 1, the vibration value of each measuring point of the overhung rotor-bearing system was small, and the vibration values of the axial measuring points of the two bearing seats (measurement points 3 and 8) were larger than those in other directions. Under condition 2, the vibration values at measurement points 1 to 4 of the drive-end bearing seat were significantly larger than those under normal conditions, especially at measurement point 4. In contrast, the vibration values at different measurement points on the free-end bearing seat were relatively similar to those under condition 1. Under condition 3, the axial vibration values of the two bearing seats shown at measurement points 3 and 8 were larger. Under the compound fault reflected in condition 4 in Table 1, not only the axial vibration values of the two bearing seats shown at measurement points 3 and 8 were large, but also the vibration of the loosened anchor bolt of the drive-end bearing seat shown at measurement point 4 was significantly greater than that of the normal free-end bearing seat shown at measurement point 9. Under condition 5, the axial vibration value of the free-end bearing seat shown at measurement point 8 was obviously greater than that of the drive-end bearing seat shown at measurement point 3, while the vibration values of other measurement points were similar to that in condition 1. However, uncertainty existed in the measurement points (such as point 1, point 2 and point 7) under the four fault conditions, and the vibration values under some conditions were relatively large, which did not reveal the cause of the large vibration of the drive-end bearing seat only from the vibration values. 

### 4.2. Results and Analysis of the High-Speed Camera Measurement

The proposed method was used to calculate 1000 frames of the video sequence output by the video capture terminal on a notebook computer with an 8-core CPU and a 32 GB memory, and the motion magnification video was obtained in 69.82 s. The amplification factor α was uniformly set to 200 in the analysis process. Considering that the motor speed had a small fluctuation at the operating frequency in the actual measurement, and the characteristic frequencies of rotating machinery faults are usually concentrated at the 1× frequency (24.1 Hz) and 2× frequency (48.2 Hz), the specified frequency band (*w_l_*, *w_h_*) was set to 20–28 Hz and 43–53 Hz, respectively. That is, 1× frequency and 2× frequency were covered.

The spatial cutoff wavelength *λ_c_* under the five working conditions was calculated by the proposed method, respectively, as shown in Table 3. The spatial cutoff wavelength values were similar in each condition; because the video of every condition was captured in the laboratory, the camera angle and the ambient brightness did not change, which shows the robustness of the proposed method in calculating the spatial cutoff wavelength.

#### 4.2.1. Condition 2

For the video obtained by the proposed method, in order to display the features of the normal and the fault conditions in the video in the form of images, ImageJ software was used to extract the time-brightness curve of the first 100 frames of the magnified video, and the 2 frames of images were determined according to the horizontal coordinate (that is, the number of frames) corresponding to the adjacent maximum and minimum brightness values in the time-brightness curve. The same method was used for all subsequent conditions. 

**Figure 5:**
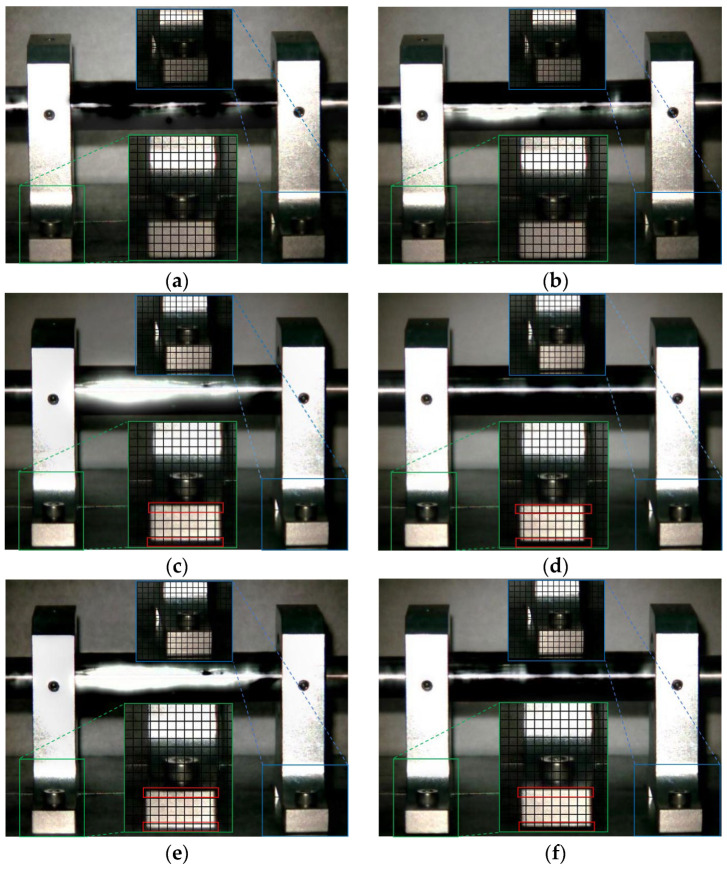
MM images of two frames at 1× frequency under condition 1 and condition 2. (**a**) Normal (maximum frame); (**b**) normal (minimum frame); (**c**) fault (maximum frame)—traditional method; (**d**) fault (minimum frame)—traditional method; (**e**) fault (maximum frame)—improved method; (**f**) fault (minimum frame)—improved method.

The Eulerian motion magnification images of the two frames at 1× frequency under the normal working condition (i.e., condition 1) and condition 2 are shown in Figure 5. As can be seen from Figure 5a,b, even if the amplification factor was 200, there was no obvious displacement change in each direction of the two bearing seats under condition 1. In contrast, the images of adjacent maximum frame (Figure 5c) and minimum frames (Figure 5d) obtained by the traditional method under condition 2 showed that the drive-end bearing seat in the green box had displacement changes only in the vertical direction, and that there was no obvious displacement in the horizontal and axial directions, while the free-end bearing seat in the blue box had no obvious displacement in all directions, as under condition 1. The displacement variation characteristics of Figure 5e,f obtained by the improved method under condition 2 were consistent with those of Figure 5c,d obtained by the traditional method, but the variation amplitude of the former was significantly greater than that of the latter. The Eulerian motion magnification video and the two frames of images reflecting the vibration of the bearing seats captured under the two conditions were consistent with the vibration values of 10 measurement points in the corresponding condition in Table 2, which indicates the accuracy of the proposed method.

**Figure 6:**
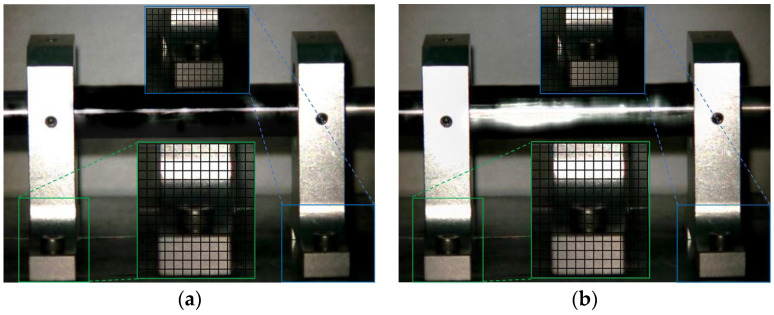

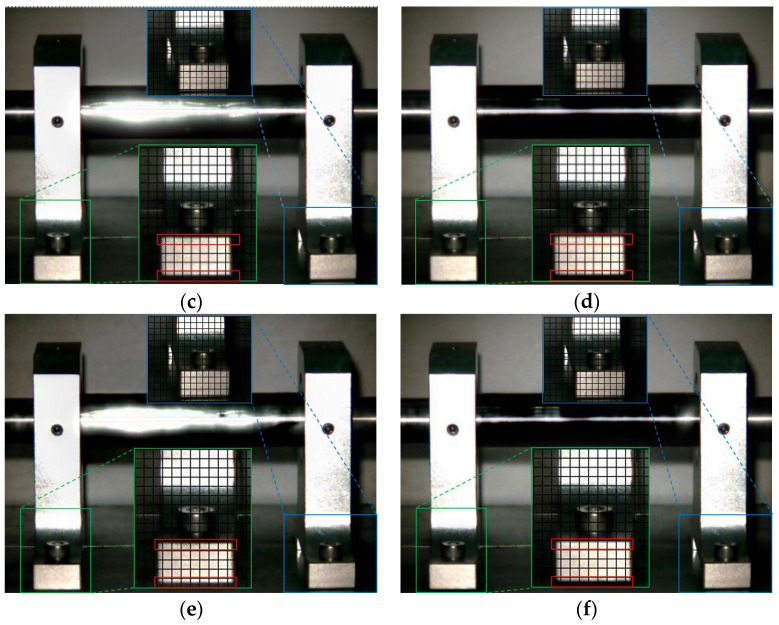
MM images of two frames at 2× frequency under condition 1 and condition 2. (**a**) Normal (maximum frame); (**b**) normal (minimum frame); (**c**) fault (maximum frame)—traditional method; (**d**) fault (minimum frame)—traditional method; (**e**) fault (maximum frame)—improved method; (**f**) fault (minimum frame)—improved method.

The Eulerian motion magnification images of the two frames at 2× frequency under condition 1 and condition 2 are shown in Figure 6. As can be seen from Figure 6, the image features of 2× frequency under condition 1 and 2 are the same as those in Figure 5, respectively.

#### 4.2.2. Condition 3

**Figure 7:**
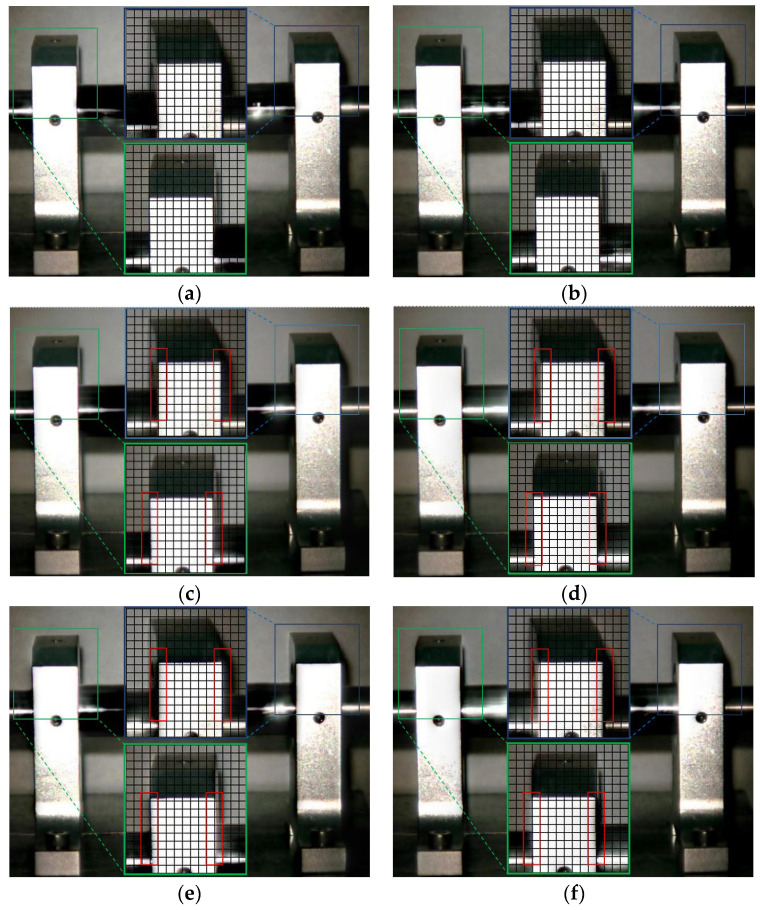
MM images of two frames at 1× frequency under condition 1 and condition 3. (**a**) Normal (maximum frame); (**b**) normal (minimum frame); (**c**) fault (maximum frame)—traditional method; (**d**) fault (minimum frame)—traditional method; (**e**) fault (maximum frame)—improved method; (**f**) fault (minimum frame)—improved method.

The Eulerian motion magnification images of the two frames at 1X frequency under condition 1 and condition 3 are shown in Figure 7. As can be seen from Figure 7a,b, even if the amplification factor was 200, there was no obvious displacement change in each direction of the two bearing seats under condition 1. In contrast, the images of the adjacent maximum frame (Figure 7c) and minimum frame (Figure 7d) obtained by the traditional method under condition 3 show that the two bearing seats in the all boxes had displacement changes in the axial direction, which indicates that when the fly wheel of the overhung rotor-bearing system in the paper had an unbalanced rotor fault, the two bearing seats vibrated axially under the action of unbalanced forces. The displacement variation characteristics of Figure 7e,f obtained by the improved method under condition 3 were consistent with those of Figure 7c,d obtained by the traditional method, but the variation amplitude of the former was significantly greater than that of the latter. The Eulerian motion magnification video and the two frames of images reflecting the vibration of the bearing seats captured under the two conditions were consistent with the vibration values of 10 measurement points in the corresponding condition in Table 2, which indicates the accuracy of the proposed method.

**Figure 8:**
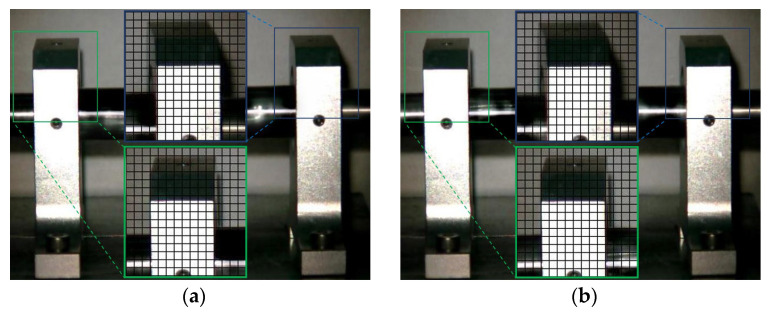

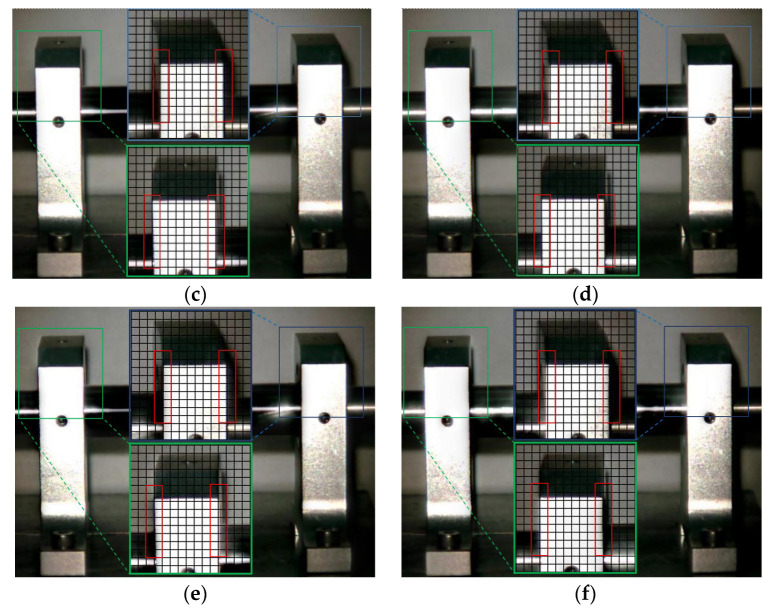
MM images of two frames at 2× frequency under condition 1 and condition 3. (**a**) Normal (maximum frame); (**b**) normal (minimum frame); (**c**) fault (maximum frame)—traditional method; (**d**) fault (minimum frame)—traditional method; (**e**) fault (maximum frame)—improved method; (**f**) fault (minimum frame)—improved method.

The Eulerian motion magnification images of the two frames at 2× frequency under condition 1 and condition 3 are shown in Figure 8. As can be seen from Figure 8, the image features of 2× frequency under condition 1 and 3 were the same as those in Figure 7, respectively.

#### 4.2.3. Condition 4

**Figure 9:**
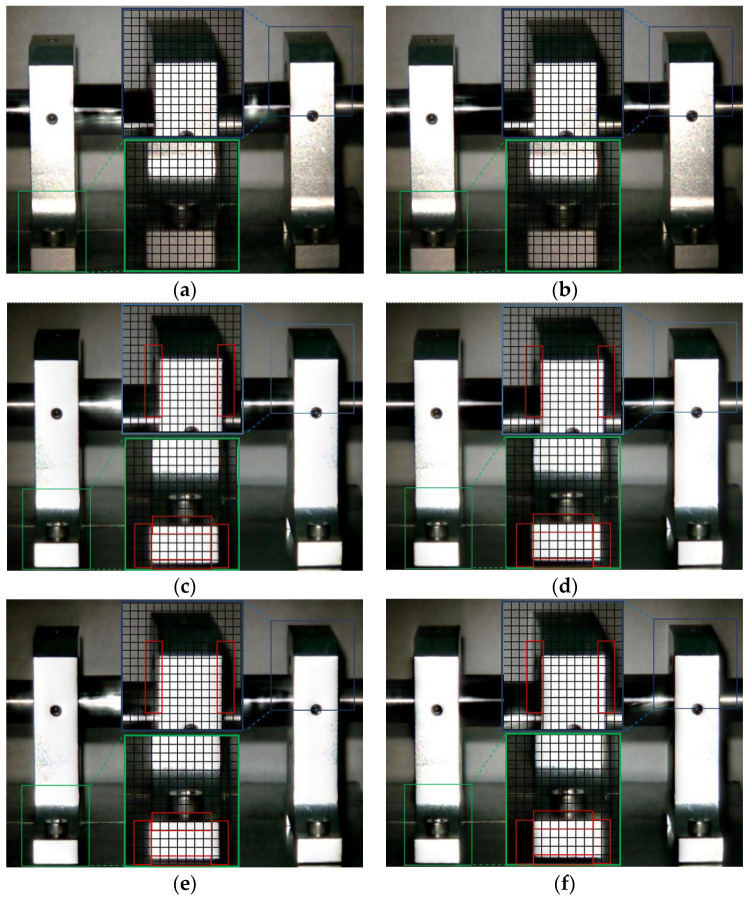
MM images of two frames at 1× frequency under condition 1 and condition 4. (**a**) Normal (maximum frame); (**b**) normal (minimum frame); (**c**) fault (maximum frame)—traditional method; (**d**) fault (minimum frame)—traditional method; (**e**) fault (maximum frame)—improved method; (**f**) fault (minimum frame)—improved method.

The Eulerian motion magnification images of the two frames at 1× frequency under condition 1 and condition 4 are shown in Figure 9. As can be seen from Figure 9a,b, even if the amplification factor was 200, there was no obvious displacement change in each direction of the two bearing seats under condition 1. In contrast, the images of adjacent maximum frame (Figure 9c) and minimum frame (Figure 9d) obtained by the traditional method under condition 4 show that the bottom of the drive-end bearing seat in the green box had displacement changes in both vertical and axial directions, while the upper part of the free-end bearing seat in the blue box only had axial displacement, which reflected the fault features of condition 2 in Section 4.2.1 and of condition 3 in Section 4.2.2. The displacement variation characteristics of Figure 9e,f obtained by the improved method under condition 4 were consistent with those of Figure 9c,d obtained by the traditional method, but the variation amplitude of the former was significantly greater than that of the latter. The Eulerian motion magnification video and the two frames of images reflecting the vibration of the bearing seats captured under the two conditions were consistent with the vibration values of 10 measurement points in the corresponding condition in Table 2, which indicates the accuracy of the proposed method.

**Figure 10:**
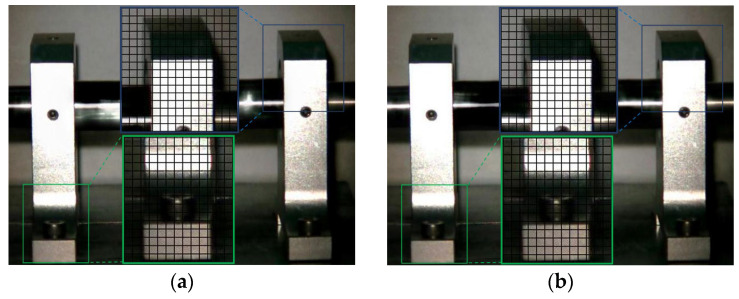

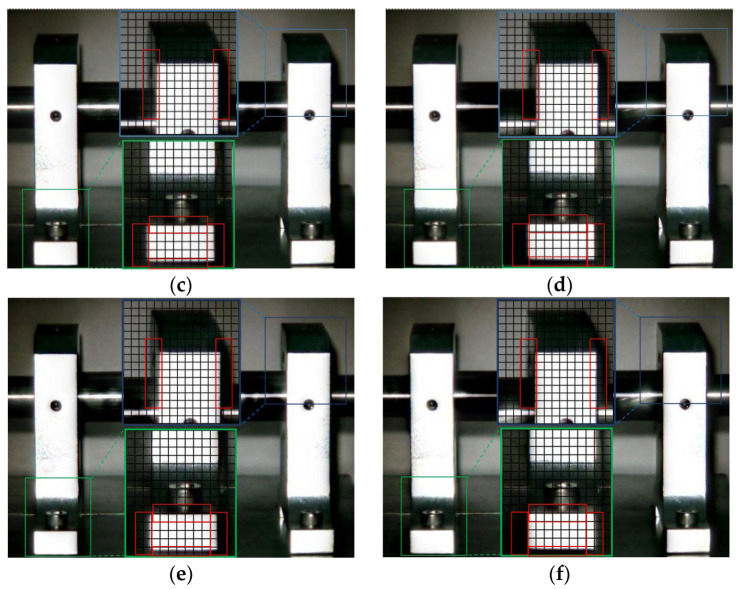
MM images of two frames at 2× frequency under condition 1 and condition 4. (**a**) Normal (maximum frame); (**b**) normal (minimum frame); (**c**) fault (maximum frame)—traditional method; (**d**) fault (minimum frame)—traditional method; (**e**) fault (maximum frame)—improved method; (**f**) fault (minimum frame)—improved method.

The Eulerian motion magnification images of the two frames at 2× frequency under condition 1 and condition 4 are shown in Figure 10. As can be seen from Figure 10, the image features of 2× frequency under condition 1 and condition 4 were the same as those in Figure 9, respectively.

#### 4.2.4. Condition 5

**Figure 11:**
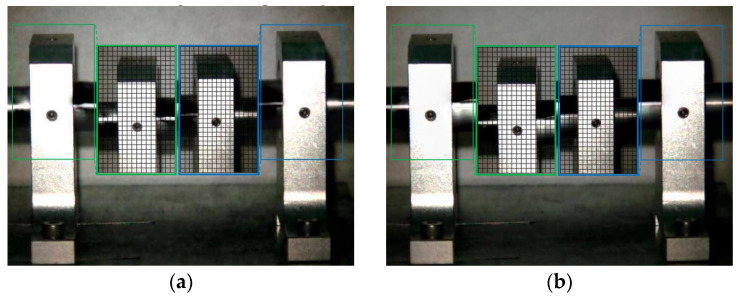

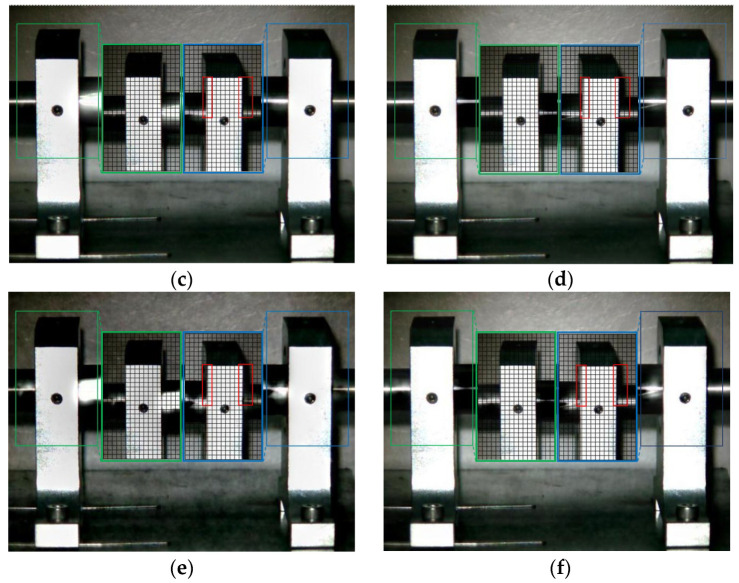
MM images of two frames at 1× frequency under condition 1 and condition 5. (**a**) Normal (maximum frame); (**b**) normal (minimum frame); (**c**) fault (maximum frame)—traditional method; (**d**) fault (minimum frame)—traditional method; (**e**) fault (maximum frame)—improved method; (**f**) fault (minimum frame)—improved method.

The Eulerian motion magnification images of the two frames at 1× frequency under condition 1 and condition 5 are shown in Figure 11. As can be seen from Figure 11a,b, even if the amplification factor was 200, there was no obvious displacement change in each direction of the two bearing seats under condition 1. In contrast, the images of adjacent maximum frame (Figure 11c) and minimum frame (Figure 11d) obtained by the traditional method under condition 5 show that the driving-end bearing seat in the green box had no obvious displacement change, while the free-end bearing seat in the blue box had axial displacement. The displacement variation characteristics of Figure 11e,f obtained by the improved method under condition 5 were consistent with those of Figure 11c,d obtained by the traditional method, but the variation amplitude of the former was significantly greater than that of the latter. The Eulerian motion magnification video and the two frames of images reflecting the vibration of the bearing seats captured under the two conditions were consistent with the vibration values of 10 measurement points in the corresponding condition in Table 2, which indicates the accuracy of the proposed method.

**Figure 12:**
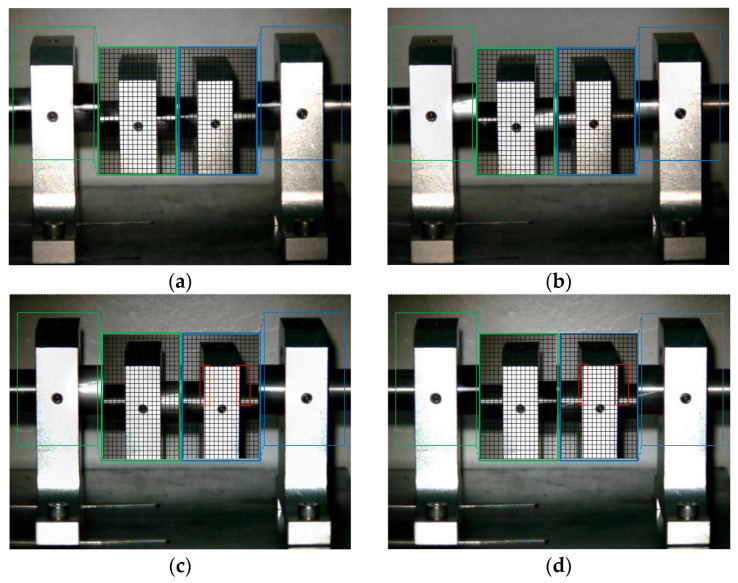

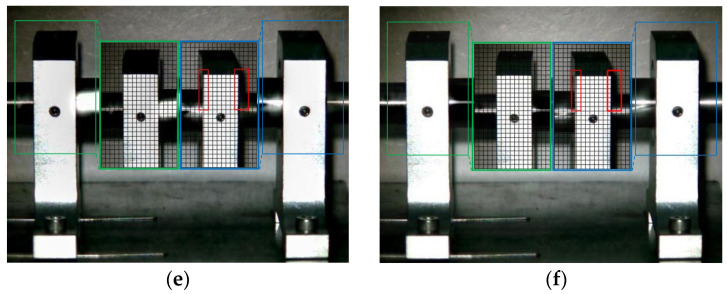
MM images of two frames at 2× frequency under condition 1 and condition 5. (**a**) Normal (maximum frame); (**b**) normal (minimum frame); (**c**) fault (maximum frame)—traditional method; (**d**) fault (minimum frame)—traditional method; (**e**) fault (maximum frame)—improved method; (**f**) fault (minimum frame)—improved method.

The Eulerian motion magnification images of the two frames at 2× frequency under condition 1 and condition 5 are shown in Figure 12. As can be seen from Figure 12, the image features of 2× frequency under condition 1 and condition 4 were the same as those in Figure 11, respectively.

## 5. Conclusions

Based on the analysis of the Eulerian motion magnification method, this paper emphasized the importance of spatial cutoff wavelength to the quality of motion magnification image. In view of the defects that can arise when obtaining the parameters manually, combined with the characteristics of HSV color space, an automatic calculation method of spatial cutoff wavelength was proposed. The publicly available video was used for the simulation analysis, and an overhung rotor-bearing experimental device was used for the experimental verification. The main research results are as follows:

(1) Using a sourced publicly available video for the simulation analysis, the proposed automatic calculation method of spatial cutoff wavelength based on HSV color space had strong robustness, and the obtained motion magnification image could better reflect the small motion, which solved the problem of defects arising from the manual parameter adjustment of the current Euler motion magnification method.
(2) Using the improved method, the overhung rotor-bearing test device had no obvious displacement change in the passband region covering 1× frequency and 2× frequency under the normal working condition.
(3) By using the improved method, it can be shown that in the passband region covering 1× frequency and 2× frequency of the overhung rotor-bearing test device, the drive-end bearing seat with the loosened anchor bolt fault had an obvious displacement change in the vertical direction, while the bearing seat without the fault had no obvious displacement in each direction. There were obvious axial displacement changes in both bearing seats under the unbalanced condition. Under the compound fault condition, the motion magnification video comprehensively reflected the single fault characteristics of each condition. Under the misalignment fault condition, axial displacement existed in the free-end bearing seat, but no significant displacement existed in the drive-end bearing seat.
(4) The improved Eulerian video motion magnification method could not only visually diagnose the common faults of rotating machinery, but could also determine the fault location, which provides a new method for the non-contact fault diagnosis of rotating machinery.

However, the method was still based on the Eulerian motion magnification. If the ambient brightness was insufficient when the video was captured, it was necessary to use a no-strobe light, so as to reduce the influence of ambient brightness changes on the analysis results. The method mainly reflected the translational displacement of the measured object, and the effect of torsional deformation was not good. The cut-off frequency band needed to be selected based on prior knowledge. Since the method could reveal the subtle structural vibration, it has potential applications in deformation monitoring, blade damage detection, machinery fault diagnosis and other fields in future works.

## Figures and Tables

**Table 1:** Five conditions and descriptions.

Name	Descriptions
Condition 1	Normal.
Condition 2	Loosen one of the anchor bolts of the drive-end bearing seat.
Condition 3	Fix 13 g bolts at the fly wheel.
Condition 4	Set to the compound fault of condition 2 and condition 3.
Condition 5	A 0.5 mm thickness gasket is arranged under the drive-end bearing seat.

**Table 2:** Vibration value of each measurement point under five working conditions. (Unit: mm/s).

Mesurement Point	Condition 1	Condition 2	Condition 3	Condition 4	Condition 5
1	0.24	2.04	1.86	3.23	0.77
2	0.35	1.26	0.34	1.12	0.3
3	0.70	1.22	1.71	1.50	1.28
4	0.22	3.6	0.42	2.48	0.21
5	0.27	0.16	0.26	0.23	0.2
6	0.35	0.29	0.52	0.48	0.54
7	0.23	0.24	1.9	2.0	0.61
8	1.04	0.67	1.71	1.12	2.94
9	0.26	0.27	0.41	0.5	0.36
10	0.29	0.24	0.46	0.4	0.33

**Table 3:** Spatial cutoff wavelength values under five conditions.

Condition	1	2	3	4	5
*λ_c_*	51.28	51.48	56.20	58.54	51.92

## Data Availability

Data are contained within the article.

## Supplementary Materials

The following supporting information can be downloaded at https://www.mdpi.com/1424-8220/23/23/9582/s1.
